# The Impact of Resistance Exercise Program on Muscle Strength, Functional Performance, and Quality of Life in Patients with Rheumatologic Disease Receiving High-Dose Glucocorticoids Treatment: A Randomized Trial

**DOI:** 10.5152/ArchRheumatol.2026.10964

**Published:** 2026-01-16

**Authors:** Meltem Karacaatlı, Kerem Abacar, Murat Karabacak, İlker Yağcı, Fatma Alibaz Öner, Haner Direskeneli, Özge Keniş Coşkun

**Affiliations:** 1 Department of Physical Medicine and Rehabilitation, Health Sciences University Medical Faculty, Kartal Research and Training Hospital, İstanbul, Türkiye; 2 Department of Rheumatology, Marmara University Medical Faculty, İstanbul, Türkiye; 3 Department of Physical Medicine and Rehabilitation, Marmara University Medical Faculty, İstanbul, Türkiye

**Keywords:** Corticosteroid, exercise, muscle strength, myopathy

## Abstract

**Background/Aims:**

This study aimed to evaluate the effectiveness of therapeutic exercises in improving muscle strength, functional status, and quality of life in patients receiving high-dose glucocorticoid therapy, which is known to cause muscle weakness and atrophy.

**Materials and Methods:**

This randomized, controlled, single-center study included 40 participants aged 18-65 years who received high-dose glucocorticoids due to chronic rheumatologic conditions, and the patients were divided into 2 groups: an exercise group and a control group. The exercise group performed lower-extremity resistance exercises for 3 months. The exercise program consisted of squats, seated leg extensions, seated leg curls, hip abductions, and seated calf raises performed 5 days per week. Functional assessments included the 6-minute walk test, the Five Times Sit To Stand Test, and the Timed Up and Go Test. Muscle strength was measured using a dynamometer, quality of life was assessed with the Short Form Health Survey questionnaire, and the cross-sectional area (CSA) of the rectus femoris muscle was evaluated via B-mode ultrasound. Patients were assessed at baseline and after 3 months of treatment.

**Results:**

Twenty patients were included in each group, with no significant differences in baseline characteristics. Compared with the control group, a significant increase was observed in knee extension strength (*P* = .00 for right and left knees) and quality of life in terms of physical function in the exercise group. However, no significant differences were found in the 6-minute walk test, the Five Times Sit To Stand Test, the Timed Up and Go Test, or the rectus femoris muscle CSA.

**Conclusion:**

In patients with rheumatologic diseases treated with high-dose glucocorticoids, resistance exercise therapy can increase knee extension strength and quality of life in terms of physical function.

**Main Points**

- Resistance exercise significantly improves knee extensor muscle strength in patients with rheumatologic diseases receiving high-dose glucocorticoids.
- The exercise program enhances quality of life, particularly in the physical functioning domain.
- Despite improvements in muscle strength, resistance exercise did not lead to significant changes in functional performance tests or muscle cross-sectional area.

## Introduction

Glucocorticoid-induced myopathy, first described by Harvey Cushing in 1932, is a well-known side effect of glucocorticoid therapy, characterized by fatigue and progressive muscle weakness.^1^ Although glucocorticoid-induced myopathy can present acutely, the chronic form is more common, typically manifesting as slowly progressive proximal muscle weakness, particularly in the lower limbs.^2,3^ Patients often report painless difficulty with activities like climbing stairs or rising from a chair, and atrophy may develop over weeks to months.

The incidence of glucocorticoid-induced myopathy is 50%-60% in long-term corticosteroid users.^4,5^ Higher doses (>10 mg/day prednisone equivalent) and fluorinated glucocorticoids (e.g., dexamethasone) pose greater risks.^6,7^ Systemic administration is most commonly implicated, though rare cases occur after inhaled or epidural use.^8,9^ Additional risk factors for the development of steroid myopathy include advanced age, malignancy, and physical inactivity.^4^ Glucocorticoids cause muscle atrophy through anti-anabolic and catabolic effects, including inhibition of amino acid transport into muscle cells, suppression of satellite cell differentiation, reduced insulin-like growth factor-1 synthesis, and accelerated breakdown of myofibrillar proteins.^10^ There is no specific diagnostic test for glucocorticoid-induced myopathy. The diagnosis is mostly based on history and physical examination. Management focuses on minimizing glucocorticoid exposure, and muscle strength often improves after discontinuation.^11^ For patients requiring ongoing therapy, switching to non-fluorinated steroids may reduce risk.^12^

The role of exercise in mitigating glucocorticoid-induced myopathy remains unclear. Physical therapy may be beneficial in preventing and treating muscle weakness in patients receiving glucocorticoids.^13,14^ But another study shows that high-intensity exercise may delay recovery.^15^ Given this inconsistency, the aim was to evaluate whether a structured therapeutic exercise program incorporating these evidence-based approaches, when initiated alongside glucocorticoid therapy, improves muscle strength and quality of life in affected patients.

## Materials and Methods

This prospective randomized controlled trial was conducted between 2019 and 2022 in accordance with the Declaration of Helsinki, after receiving ethics approval from Marmara University Faculty of Medicine on February 1, 2019 (approval number: 09.2019.156). All participants provided written and verbal informed consent before enrollment.

The study included patients aged 18-65 years with rheumatologic disease, including Behçet’s disease, systemic vasculitis, or glomerulonephritis, who were initiating glucocorticoid treatment at a dose of 32 mg/day or higher at Marmara University’s Rheumatology Department. Exclusion criteria were: history of neuromuscular disease; poor general condition preventing independent ambulation; cardiopulmonary disease; active infection; major surgery within the previous 6 months; or refusal to participate in the study. A simple randomization method (via a web-based system) was used to allocate patients to groups. After patients were randomized, their demographic data were recorded by the first clinician, and they were referred to a physiotherapist for exercise training in a sealed envelope. Physical and ultrasonographic evaluations were performed by a second clinician, blinded to clinical and group information.

The exercise intervention group performed a resistance training program using elastic bands that targeted the proximal muscles of the lower extremities, beginning at the initiation of glucocorticoid treatment for 3 months. The exercise program consisted of squats, seated leg extensions, seated leg curls, hip abductions, and seated calf raises performed 5 days per week. Each session included 3 sets of 15 repetitions of each exercise, preceded and followed by 5-minute warm-up and cool-down periods. The exercise program was continued for 3 months without any changes in the exercise plan. Patients received detailed exercise guides with illustrations and maintained exercise diaries. Patients were called every 2 weeks to check their compliance with the exercise program. The control group had no exercise program or sports activity other than daily living activities.

**Figure 1.**
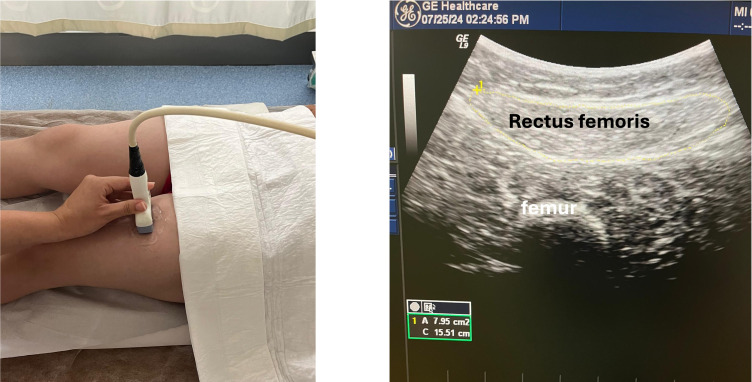
The position of the probe and the visualization of rectus femoris muscle for measurement.

The primary outcome was to evaluate muscle strength and the patient’s functional status, while the secondary outcome was to evaluate the rectus femoris cross-sectional area (CSA) and the patient’s quality of life. Knee extensor muscle strength was measured using a dynamometer while the patient was seated on the examination table and the hip and knee were flexed at 90 degrees. The dynamometer was placed in the middle of the tibia, and the patient extended the knee. The average of the 3 measurements was recorded as the final value. Functional assessments included the 6-minute walk test which is conducted in a 30-meter corridor after 15 minutes of rest; the Timed Up and Go Test which measures the time to stand from a chair, walk 3 meters, and return to sitting; and the Five Times Sit To Stand Test which records the time required to sit down and stand up from a chair 5 consecutive times. Quality of life was evaluated using the 36-item Short Form Health Survey (SF-36) questionnaire. Rectus femoris muscle CSA was measured bilaterally using B-mode ultrasound (Esaote MyLab Six) by a single clinician experienced in musculoskeletal ultrasonography. Measurements were taken at the midpoint of the thigh between the anterior superior iliac spine and the superior patellar border with patients in the supine position and knees fully extended, ensuring minimal probe pressure and muscle relaxation during imaging. The position of the ultrasound probe and the ultrasonographic image are shown in Figure 1. Outcome measurements were performed at baseline and at the third month of treatment by a clinician blinded to patient group information.

## Results

**Figure 2.**
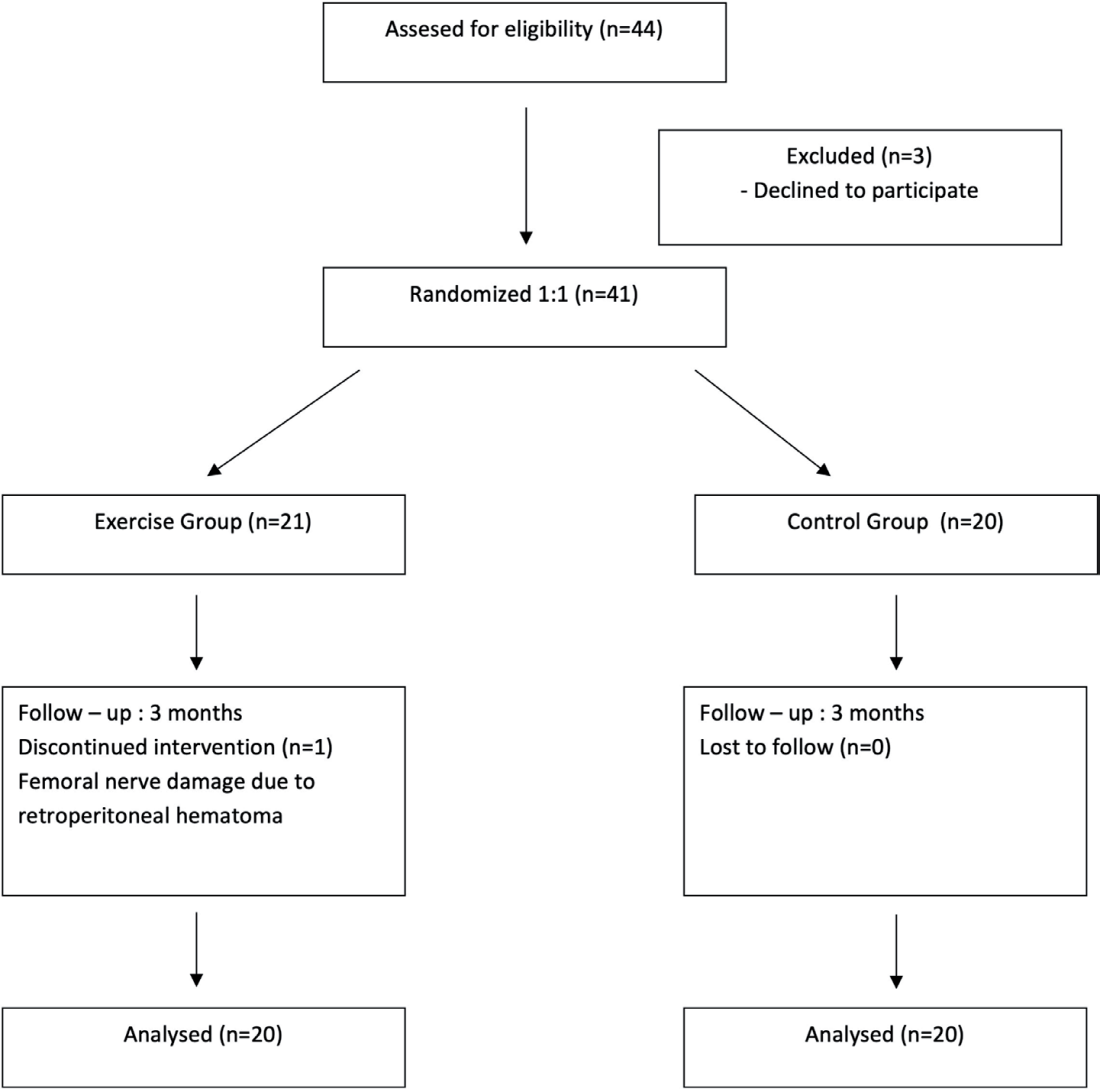
Patient flowchart.

Twenty participants were systematically allocated to each group, comprising 11 females and 9 males in the exercise group, and 10 females and 10 males in the control group. There was only 1 dropout in the patient who developed femoral nerve damage following retroperitoneal bleeding. The flow chart is presented in Figure 2.

The mean age of individuals in the exercise group was 40.7, while it was 36.5 in the control group. No significant difference was observed between the 2 groups in demographic parameters (*P* > .05). Demographic data are shown in Table 1.

The mean initial glucocorticoid dose administered was 42.8 mg in the exercise group and 47.6 mg in the control group, and no statistically significant difference was observed between the 2 groups in terms of the start of glucocorticoid treatment (*P* = .082). At the 3-month interval, the mean steroid dosages were 3.7 mg and 6.1 mg in the exercise and control groups, respectively. Preceding the initiation of glucocorticoid treatment, the right rectus femoris CSA measured 9.2 cm^2^ in the exercise group and 9.0 cm^2^ in the control group, while the left rectus femoris CSA was 9.2 cm^2^ in the exercise group and 9.1 cm^2^ in the control group. No statistically significant difference was observed between the right and left rectus femoris CSAs across the groups (*P* = .86 for the right, *P* = .92 for the left).

In the exercise group, there were no statistically significant alterations noted in the rectus femoris CSAs, the Five Times Sit To Stand Test, the Timed Up and Go Test, and the 6-minute walk test before and after the treatment (*P* > .05). However, a significant enhancement in knee extension strength was evident for both legs following the implementation of the resistance exercise program. Specifically, the right knee extension strength increased from 9.8 kg before treatment to 11.4 kg after treatment (*P* = .01), and the left knee extension strength increased from 9.4 kg before treatment to 10.0 kg after treatment (*P* = .02).

In the control group, no statistically significant differences were discerned in the rectus femoris CSAs, the Five Times Sit To Stand Test, the Timed Up and Go Test, the 6-minute walk test, and knee extension strengths. Nevertheless, a non-statistically significant decrease in knee extension strength was observed post-glucocorticoid treatment, with right knee extension registering 9.98 kg before treatment and 8.63 kg after treatment (*P* = .885), and left knee extension measuring 8.43 kg before treatment and 8.09 kg after treatment (*P* = .174).

Upon comparison of the exercise and control groups, no statistically significant distinctions were noted in the rectus femoris CSAs, the Five Times Sit To Stand Test, the Timed Up and Go Test, and the 6-minute walk test (*P* > .05). However, a statistically significant elevation in knee extension strength was identified in the exercise group following steroid treatment in comparison to the control group. Specifically, the right knee extension strength was 11.48 kg in the exercise group, contrasting with 8.63 kg in the control group (*P* = .00), and the left knee extension strength was 10.04 kg in the exercise group, in contrast to 8.09 kg in the control group (*P* = .00). Muscle strength, functional assessment, and rectus femoris CSA data are shown in Table 2.

As a result of the comparative analysis of the Quality of Life SF-36 index scores between the groups in the third month of treatment, significant differences were detected only in the physical function subparameter. Specifically, the physical function score exhibited a statistically significant elevation within the exercise group as opposed to the control group (*P* = .024). Comparison of quality of life scores between groups is shown in Table 3.

## Discussion

The principal objective of this study was to investigate the preventive effects of exercise on the decline in muscle strength in individuals afflicted with rheumatologic disease who had undergone treatment with high doses of glucocorticoids. In the current investigation, the findings elucidate that the implementation of an exercise program resulted in a significant augmentation of knee extension strength and quality of life in terms of physical function among patients undergoing high-dose steroid therapy.

Braith et al^13^ reported that in patients receiving glucocorticoids after heart transplantation, strength in knee extension, chest press, and lumbar extension increased 4- to 6-fold in the resistance exercise program group compared to the control group. In the study conducted by Horber et al,^16^ a decrease in mid-thigh muscle CSA and a 20% decrease in mean peak torque of the quadriceps femoris muscle were observed in patients receiving glucocorticoid therapy after kidney transplantation. However, following the implementation of the exercise regimen, a significant improvement in mean peak torque and mid-thigh CSA was achieved in these patients, leading to a restoration of muscle strength comparable to the control group. These 2 studies have demonstrated that exercise can mitigate muscle weakness associated with glucocorticoid use. Nevertheless, it is worth noting that both studies were conducted exclusively in cohorts of patients receiving low-dose glucocorticoid regimens. In the present study, similar to the findings of Horber et al,^16^ the CSA of the rectus femoris increased by 11% on the right and 9% on the left in the exercise group, although not statistically significant.

A study conducted by Nagashima et al^17^ revealed a significant 10% decrease in knee extension strength in connective tissue disease patients undergoing exercise therapy while simultaneously receiving high-dose glucocorticoid therapy. This study did not include a control group, so data from another study of patients with interstitial lung disease receiving high-dose glucocorticoid therapy were used for muscle strength comparison. This study showed a significant 28% decrease in knee extension strength and a significant 17% decrease in handgrip strength after glucocorticoid therapy.^18^ In the study conducted by Nagashima et al,^17^ indirect evidence has shown that muscle strength loss can be reduced with an exercise program. In the present study, a 13% decrease in right knee extensor muscle strength was observed in the control group, similar to other studies, while a significant increase in knee extension strength was observed in the exercise group. In the study by Nagashima et al,^17^ the glucocorticoid dose was 28 mg/day, while in this study, the mean glucocorticoid dose at the time of the second evaluation was 3 mg/day in the exercise group and 6 mg/day in the control group. This difference in muscle extension strength results may be due to differences in the administered glucocorticoid doses. Although there is evidence that exercise can alleviate glucocorticoid-induced muscle weakness, exercise intensity remains unclear. A rodent study examining the effect of exercise intensity on muscle physiology found that the group receiving both intense exercise and steroid treatment had lower body weight compared to the group receiving steroids alone.^15^ Furthermore, the CSA of muscle fibers in both the soleus and extensor digitorum longus muscles was observed to be relatively smaller in the intense exercise group compared to the control group. Therefore, further studies are needed to clarify the intensity of the exercise program that should be given to patients receiving glucocorticoid treatment.

The secondary aim in this study was to evaluate the effects of high-dose glucocorticoids on activities of daily living (ADL) and muscle CSA. Hanada et al,^18^ in a study of patients with interstitial lung disease receiving glucocorticoids, observed that quadriceps femoris and handgrip strength decreased in the steroid group compared to the control group, while the 6-minute walk test, ADL, and SF-36 Quality of Life scores were similar in both groups. Consistent with these previous observations, this study revealed similar results between the exercise and control groups for the 6-minute walk test, the Five Times Sit To Stand Test, and the Timed Up and Go Test. However, in this study, a significant increase in quality of life with exercise was observed in terms of physical function. In addition, although there was no statistically significant difference in the Five Times Sit To Stand Test in this study, a 0.5-second improvement was observed in the exercise group, and a 1.05-second deterioration was observed in the control group. There were no statistically significant differences in physical assessment other than muscle strength, but changes in muscle strength and the Five Times Sit To Stand Test may have impacted patients’ quality of life.

A study by Hosono et al^19^ observed a 28% decrease in mid-thigh muscle CSA following glucocorticoid treatment in individuals with rheumatic diseases. In a study conducted by Nawata et al,^20^ paraspinal muscle volume was systematically examined using computed tomography after treatment with 0.7 mg/kg/day prednisolone. The results of the study revealed a significant decrease in muscle mass following glucocorticoid treatment.^20^ A study by Yoshido et al^21^ in a cohort of 5 healthy dogs observed a significant 9.3% decrease in mid-thigh muscle CSA following 4 weeks of prednisone treatment at a dose of 1 mg/kg per day. Unlike other studies, in this study, no decrease in muscle CSA was detected in the control group. This may be due to the low glucocorticoid dose received by the patients during the second evaluation. However, in the exercise group, an 11% increase in the right rectus femoris CSA and a 9% increase in the left rectus femoris CSA were observed, although not statistically significant.

This study has some limitations. The patient group in the study was heterogeneous. However, it is difficult to create a homogeneous patient group, and similar heterogeneity has been observed in previous studies. Because the exercise protocol was administered as a home program, patient compliance with the program could be considered an additional constraint. However, adherence to the program was assessed using the patient’s exercise diary, and patients were contacted biweekly to ensure adherence. Another limitation of this study is the subjective nature of the exercise intensity assessment. In this study, the CSA of the rectus femoris muscle was measured at mid-thigh. Body mass index–adjusted CSA may be used in future studies.

In conclusion, this study found that resistance exercise therapy improved knee extension strength and quality of life in terms of physical function in patients treated with high-dose glucocorticoids. Therefore, a home-based exercise program may be recommended for patients receiving high-dose glucocorticoids.

## Figures and Tables

**Table 1.** Demographics Data

	Exercise Group		Control Group		*t*	*P*
	Mean	SD	Mean	SD		
Age (years)	40.7	12.6	36.5	11.1	1.09	.27
Height (cm)	166.2	7.8	168.1	8.0	0.77	.44
Weight (kg)	69.5	14.12	70.9	9.98	0.362	.38
BMI (kg/m^2^)	25.18	5.19	25.13	3.5	0.033	.291

SD, standard deviation; BMI, body mass index.

**Table 2.** Muscle Strength, Functional Assessment, and Rectus Femoris Cross-Sectional Area Outcomes

	Exercise Group			Control Group			Comparison Between Groups	
	Before Treatment	After Treatment	*P*	Before Treatment	After Treatment	*P*	*t*	*P*
CSA-R (cm^2^)	9.22 ± 3.07	10.37 ± 3.38	.47	9.07 ± 2.62	9.07 ± 2.02	0.868	1.476	.148
CSA-L (cm^2^)	9.22 ± 2.75	10.11 ± 3.19	.53	9.14 ± 2.76	9.49 ± 1.97	0.922	0.738	.465
Five Times Sit To Stand Test (sec)	9.81 ± 2.12	9.34 ± 1.73	.85	9.13 ± 2.80	10.15 ± 1.49	0.391	1.597	.119
Timed Up and Go Test (sec)	6.32 ± 1.44	5.94 ± 0.78	.93	6.5 ± 1.39	6.33 ± 1.10	0.699	1.19	.241
6-Minute Walk Test (m)	608 ± 46	612 ± 51	.79	611 ± 53	610 ± 48	0.442	0.906	.371
Knee extension-R (kg)	9.8 ± 2.53	11.4 ± 2.15	.01*	9.98 ± 1.81	8.63 ± 1.33	0.885	5.026	0*
Knee extension-L (kg)	9.4 ± 2.73	10.0 ± 2.06	.02*	8.43 ± 1.60	8.09 ± 1.03	0.174	3.776	0*

CSA, cross-sectional area; L, left; R, right.

**Table 3.** Comparison of Quality of Life Scores Between Groups

	Control Group		Exercise Group		*P*
	Mean	SD	Mean	SD	
Physical function	84.00	9.94	90.75	8.15	.024*
Limitation due to physical health	68.75	33.31	73.75	31.90	.631
Limitation due to emotional problems	61.66	43.63	66.66	41.88	.714
Energy-fatigue	45.00	12.24	46.25	13.16	.758
Emotional well-being	71.20	5.59	69.20	7.68	.353
Social function	58.75	13.51	68.12	11.08	.21
Pain	76.77	16.60	82.37	14.92	.269
General health	38.00	9.51	32.25	10.32	.386

SD, standard deviation

## Data Availability

The data that support the findings of this study are available on request from the corresponding author.
